# Strengthening Newborn Screening, Diagnosis and Management of Birth Defects in Low- and Middle-Income Countries: A Priority for WHO Member States

**DOI:** 10.3390/ijns12040075

**Published:** 2026-09-23

**Authors:** Ayesha De Costa, Pablo Duran, Rajesh Khanna, Janet Kayita, Khalid Siddeeg

**Affiliations:** 1 Newborn Health, WHO HQ Geneva, 1211 Geneva, Switzerland; 2 Perinatal Health at the Pan American Health Organization, Washington, DC 20037, USA; duranpa@paho.org; 3 Newborn, Child & Adolescent Health, WHO Regional Office for Southeast Asia, New Delhi 110002, India; khannar@who.int; 4 Family and Reproductive Health, WHO Regional Office for Africa, Brazzaville P.O. Box 06, Congo; kayitaj@who.int; 5 Child and Adolescent Health, WHO Regional Office for the Eastern Mediterranean, Cairo 11371, Egypt; siddeegk@who.int

**Keywords:** neonatal screening, congenital abnormalities, developing countries, health services accessibility, delivery of health care, integrated

## Abstract

As infectious causes of child mortality decline, birth defects represent a growing relative proportion of neonatal and under-five deaths (as well as unmeasured morbidity) in low- and middle-income countries (LMICs), underscoring the need to integrate their prevention, detection and care into child health agendas. In response to World Health Assembly resolution WHA77.5, the World Health Organization (WHO) convened global consultations with LMIC programme leaders, experts, academics, civil society, affected persons and families to develop a framework for integrating newborn screening, diagnosis and management of birth defects into national health systems. This communication summarizes key lessons from country experience and sets out the core principles for implementation captured in the framework: start with one or a few feasible, high-impact conditions; link screening to diagnosis, management and long-term follow-up; assess health-system readiness before scale-up; secure sustainable financing; strengthen data systems; and provide family-centred support. The recent WHO report ‘Strengthening Newborn Screening, Diagnosis and Management of Birth Defects’ focused on LMICs and offers a pragmatic pathway for countries to expand newborn screening equitably while building the services needed to improve survival, functioning and quality of life for children with congenital conditions.

## 1. Introduction

Globally, an estimated 3–6% of infants are born with serious birth defects [1] (visible structural, metabolic, endocrine, hematologic, or functional defects like hearing), i.e., approximately 8 million newborns annually [2]. Over the past two decades, remarkable progress has been made in reducing child mortality, particularly from infectious causes. As a result of this success, birth defects now account for an increasing relative proportion of neonatal and under-five deaths, as well as a substantial unmeasured burden of lifelong disability, especially in low- and middle-income countries (LMICs). Addressing birth defects is therefore no longer optional; it is an essential component of equitable, people-centred health systems and of the global commitment to universal health coverage.

Over 90% of children with serious birth defects are born in LMICs [3], where they face a markedly higher risk of mortality compared to those born in high-income countries with the same congenital condition. Indeed, 95% of the 400,000–500,000 annual child deaths worldwide related to birth defects occur in LMICs [3,4], reflecting persistent gaps in timely detection and care. The relative share of under-five deaths due to congenital conditions has been rising in many low-income regions: for example, in sub-Saharan Africa the proportion of under-five deaths attributed to birth defects grew from about 1% in 2000 to ~4% by 2023 and in South Asia from 3% to ~11% over the same period [5]. Mortality represents only the visible part of the burden of birth defects, with a substantially larger and often irreversible burden of unmeasured morbidity resulting from conditions that are not detected and managed early.

To address this emerging challenge, the 77th World Health Assembly (WHA) in 2024 adopted a resolution (WHA 77.5) calling on Member States to consider implementing universal newborn screening (NBS) and comprehensive care for children with birth defects [6]. In line with this mandate, WHO initiated a technical consultation series in 2024–2025 aimed at developing a country-informed framework to assist LMICs in planning and implementing NBS, diagnosis, management and care for children with birth defects, integrated into national health systems. This communication summarizes the global context and key messages captured in the WHO report, highlighting common lessons from country experiences and setting out a practical framework to assess and strengthen health-system readiness [7].

## 2. Consultative Process

From September 2024 to April 2025, WHO’s Department of Maternal, Newborn, Child and Adolescent Health and Ageing convened five rounds of technical consultations with Ministry of Health representatives from ‘frontrunner’ LMICs (those that had large scale national/subnational initiatives for one or more birth defects within their national health systems), subject matter experts, clinicians, academics, civil society groups, affected persons and families. Country presentations described national and subnational experience in initiating and scaling NBS, diagnosis and management for prioritized birth defects. Experts defined and discussed various components of the NBS system. Discussions focused on condition selection, health-system readiness, financing and governance, service delivery across levels of care, referral and data systems, rehabilitation, follow-up and family support [7].

Lessons from these consultations were synthesized into a draft framework and refined through discussion and iterative feedback from participants. The inclusion of affected persons and families helped ensure that the framework remained people-centred and attentive to stigma, communication, psychosocial support and inclusive service design [7].

## 3. Results

### 3.1. Key Messages from the Consultation Captured in the WHO Framework: Reframing Newborn Screening as an Integrated Continuum of Care

#### 3.1.1. Reframing Newborn Screening as the Start of an Integrated Continuum of Care Rather than a Method of Testing

A key outcome of the WHO consultations was the recognition that NBS should not be viewed solely as blood spot testing nor an end activity in itself. Rather, NBS was conceptualized as the first step of a comprehensive system encompassing clinical, laboratory and/or physical examinations as screens, which is then linked to diagnostic confirmation, treatment, rehabilitation, long-term follow-up and family support. This broader perspective shifts the focus from screening alone, or a particular screening method, to the creation of integrated pathways that enable children with congenital conditions to be identified early and connected to the care and services required to achieve optimal health and developmental outcomes.

#### 3.1.2. When Is the Appropriate Time for an LMIC to Begin a National Initiative on NBS, Diagnosis and Management of One or More Birth Defects?

A central message was that no single socioeconomic or health-system threshold is required before countries can begin NBS and care initiatives. Countries with widely differing infant mortality rates at the time programmes were initiated—including approximately, 12–15 per 1000 in Cuba [8] and Argentina [9], 30–40 per 1000 in the Philippines [10], India [11] and Brazil, and above 40 per 1000 in Uganda [12]—were nevertheless able to begin large scale national/sub national initiatives for birth defect when political commitment, local champions, disease-burden evidence, pilot experience or partner support created momentum. The decision to start should therefore be guided less by national income level and more by equity, feasibility and the ability to link early identification with effective care [7].

#### 3.1.3. Prioritizing Feasible, High-Impact Conditions

LMIC programmes typically began with a single condition or a small set of conditions, expanding to cover additional conditions only as diagnostic, treatment, financing and laboratory capacity increased. Common selection criteria included disease burden, availability of effective treatment, feasibility of screening and alignment with existing health-system capacity. The framework emphasizes avoiding screening for conditions where diagnosis, treatment or support cannot be provided.

Country experience illustrates this phased approach. Uganda prioritized sickle cell disease by leveraging its existing HIV testing platforms [12]; India’s RBSK programme [11] began with visible congenital disorders and simple functional tests before adding more resource-intensive conditions; and the Philippines initially screened for five metabolic and endocrine conditions before expanding its panel [10].

It is often the case that robust registries for birth defects do not exist before screening begins; it may be useful to implement well planned and executed pilot studies to confirm incidence. Where robust epidemiologic data estimating the burden of different birth defects (visible and invisible) are lacking, national obstetric, neonatal and pediatric professional organizations may provide valuable input for selecting initial priority conditions, enabling pilot initiatives and programs at scale to be planned and implemented [7].

#### 3.1.4. Ensuring the Post-Screening Continuum of Care

NBS has limited value unless it is linked to confirmatory diagnosis, treatment, rehabilitation and long-term follow-up. For each screened condition, countries should establish clear referral pathways and ensure that these services are accessible before population-wide screening is introduced. For structural congenital anomalies, this includes timely access to safe children’s surgery and anesthesia; for conditions such as clubfoot, treatment may be feasible closer to primary care using task-shifted models feasible with the Ponseti method [13] for management. For conditions like sickle cell disease and congenital hypothyroidism, this could mean ensuring key medicines like hydroxyurea and thyroxine are on the essential medicines list and accessible in health facilities. Conversely, screening for conditions requiring highly specialized diagnostics or lifelong therapy may need to be deferred until the necessary capacity is in place [7]. Meanwhile, collaborations between neighbouring states/regions may allow for access to specialty or other needed capabilities on a short- or long-term basis depending on circumstances.

Quality assurance mechanisms should also be defined across the screening-to-care pathway. The specific mechanisms and parameters measured will vary depending on the condition(s) prioritized.

### 3.2. Involving All Levels of the Health System

NBS, diagnosis and management should be organized across the health system, not concentrated only in tertiary services. Community and primary care platforms can support early identification, counselling, sample collection, referral, follow-up and family support; district and secondary facilities can provide basic diagnostics, routine management and rehabilitation; and tertiary centres can deliver complex diagnostics, pediatric surgery, specialist treatment and advanced rehabilitation. Kerala’s Hridyam programme for congenital heart disease in India illustrates this approach, linking screening with confirmatory diagnosis and management across levels of care with secondary-level echocardiography, referral and transport networks, tertiary cardiac services and a centralized digital case-management platform [14].

#### 3.2.1. Assessing Health-System Preparedness While Planning a National Initiative

The WHO framework supports countries to assess whether the required components for screening, diagnosis, referral, treatment, rehabilitation and long-term follow-up are available—or can realistically be developed—for each prioritized condition (Figure 1). Starting from the screening modality, whether biochemical, clinical or visual examination, programme planners can map the care pathway and identify gaps in the workforce, diagnostics, referral, treatment, financing, data systems and quality assurance. This assessment helps define priority investments before population-wide screening is introduced [7].

Country examples show how such readiness assessments can guide investment. In Kerala, India, planning for the Hridyam congenital heart disease programme identified gaps in timely surgery, diagnostics, specialist workforce, neonatal transport and digital case management. Addressing these gaps helped expand access to pediatric cardiac care first and illustrates how aligning screening with system strengthening can enable more effective care in resource-limited settings [7,14].

Planning to introduce or expand a newborn screening programme can expose important gaps in clinical capacity and care pathways, while also creating an opportunity to strengthen access, coordination and organization of integrated services for children.

#### 3.2.2. Disability, Rehabilitation and Developmental Support

Initiatives at scale to address birth defects should aim not only to prevent mortality, but also to reduce avoidable disability and support long-term functioning, participation and quality of life. Early identification should therefore be linked to rehabilitation, habilitation, developmental support, appropriate assistive technologies, psychosocial care, inclusive early-childhood services and disability support systems. In many LMICs these services remain fragmented or concentrated in tertiary centres; India’s District Early Intervention Centers offer a good example of integrating developmental and rehabilitation services at a secondary level within the national health system [15]. Disability-inclusive care ensures that children with congenital conditions are supported not only to survive, but also to reach their developmental potential.

#### 3.2.3. Sustainable Financing and Governance

Ensuring long-term sustainability was identified as a crucial factor for effective programmes. Governments in successful programmes gradually shifted financing from donor or out-of-pocket sources to domestic government funding and national health insurance schemes, in line with universal health coverage principles. In the Philippines, for instance, the NBS program was initially funded by user fees (550 PhP) and grant support [16]. In 2006, it was integrated into the national health insurance benefit package (500 Php), covering 90% of the screening cost. As NBS expanded, the fee increased partially, limiting participation. Full national insurance coverage of the expanded screening in 2019 resulted in expansion of newborn coverage to 29 conditions in over 7000 birthing facilities by 2023. Armenia’s universal NBS program similarly began with a five-year external funding from a Swiss grant, then transitioned year on year to full government budget support by year five [7]. Government funding is key to ensuring equity and universal health coverage.

Not all countries have or require legislation around NBS, but clear governance and strong Ministry of Health leadership have been consistently important. In the Philippines, an NBS Act enacted in 2004 [17] helped institutionalize the bloodspot NBS programme and required health institutions to provide NBS as a condition for licensure, while other countries such as India achieved substantial coverage of visible birth defects through executive policies and programmatic action without specific legislation, focusing instead on effective implementation and stakeholder buy-in. Participants emphasized that while legislation can anchor a program, this does not guarantee success without corresponding investments in financing, capacity-building, and coordination [7].

#### 3.2.4. Role of Partnerships and Multi-Sectoral Engagement

Many LMICs have found that government-led partnerships across public, private, and civil society sectors were key to expanding services and specialist capacity for the management of birth defects. Public–private partnerships (PPPs) have been used to supplement government capabilities—for example, Brazil and Kerala forged PPPs with private hospitals to increase pediatric cardiac surgery capacity for congenital heart defects [14,18], and a number of African countries have partnered with international NGOs to train providers and establish clinics for clubfoot management [13]. Government leadership is needed to coordinate such partnerships, ensure quality standards and equity, and maintain integrated care pathways across sectors. Broad stakeholder engagement (e.g., with professional associations, non-governmental organizations, parent support groups, community health workers, disability services, etc.) has been instrumental in building a support system and addressing gaps [7,14,18,19,20,21].

#### 3.2.5. Human Resources and Champions

Effective programmes invested in human resource development, from training front-line health workers (midwives, nurses, primary care providers) in identifying and referring birth defects to developing specialists and national referral hubs for complex care. In some settings, champions have played an important catalytic role in generating momentum, shaping policy or securing resources [20]. These champions may come from clinical, academic, civil society or parent networks, but they can also be situated within government, where they are able to advance the agenda through routine planning, budgeting and policy processes. The key lesson is not to rely solely on individual advocacy, but to translate such leadership into institutional commitment, public-sector stewardship and sustained integration within routine health services [7].

#### 3.2.6. Data Systems and Follow-Up

Simple, robust data systems are essential to track screening coverage, results, confirmatory diagnosis, treatment initiation, follow-up and long-term outcomes. Linking NBS data to child health records, registries or digital dashboards can reduce loss to follow-up and support programme improvement. The Philippines has established NBS continuity clinics and centralized data systems to support confirmatory testing and long-term follow-up; Uganda is developing sickle cell disease registries and patient-tracking tools to improve care continuity; and India’s RBSK programme is strengthening digital platforms to track children with birth defects across referral and follow-up pathways. However, the use of robust data systems integrated across service levels is still a challenge for many LMICs [7].

Greater documentation and publication of lessons from data systems and follow-up mechanisms are needed, not only to highlight programme achievements but also to share practical learning that can inform and strengthen newborn screening design, implementation and delivery across the global community.

#### 3.2.7. Communication with Families, Addressing Stigma and Providing Family Support

Clear, timely and sensitive communication with families is essential throughout the screening and care pathway. Programmes should define who communicates results, how counselling is provided, and what families are told about positive screens and next steps. Communication should be practical, contextualized, non-stigmatizing and designed to promote timely follow-up.

Because families of affected children may face stigma, discrimination, caregiving burdens and financial stress, public education, peer support, counselling and links to disability or social support services should accompany the technical components of NBS programmes. These elements are essential to ensure that services are people-centred, acceptable and responsive to family needs [7].

## 4. Discussion

The WHO framework emerging from these consultations offers a pragmatic approach for countries seeking to strengthen NBS, diagnosis and management of birth defects within existing health systems. Rather than positioning NBS as a stand-alone laboratory or facility-based activity, the framework emphasizes screening as one component of a broader pathway that begins with identification and extends through diagnostic confirmation, referral, treatment, rehabilitation, long-term follow-up and family support. This distinction is particularly important in LMICs, where early detection without accessible care may fail to improve outcomes and may undermine public confidence in screening initiatives.

In this framework, screening is used broadly to include physical examination, biochemical screening such as dried blood spot testing, and clinical screening tests such as pulse oximetry or newborn hearing screening. Birth defects include structural, metabolic, endocrine, hematological, genetic, chromosomal and functional conditions present at birth.

A key contribution of the framework is its emphasis on health-system readiness. Implementation should be guided by a clear understanding of what is already available, what can realistically be strengthened, and which conditions can be addressed safely and equitably within current or near-term health system capacity. This shifts the policy question from whether a country is “ready” for NBS in general, to which condition, pathway and package of services can be responsibly introduced first.

The framework also highlights the importance of integration. Birth defect services intersect with maternal and newborn care, child health, immunization, nutrition, rehabilitation, disability services, surgical care and social protection. Embedding NBS and follow-up within these platforms can improve coverage, reduce fragmentation and support continuity of care. At the same time, integration requires deliberate planning: referral pathways, workforce roles, financing arrangements, data systems and accountability mechanisms must be defined from the outset. Without these elements, screening risks becoming a vertical activity that identifies children without ensuring that they receive timely and appropriate care.

The countries’ experiences reviewed during the consultations further suggest that scale-up is most feasible when programmes begin with a limited number of high-priority conditions and expand progressively as capacity develops. This incremental approach should not be viewed as lack of ambition, but as a strategy for building sustainable services, demonstrating impact and protecting equity. For structural congenital anomalies in particular, the availability of safe surgical and anesthesia services is a critical determinant of whether early detection can translate into survival and improved function. Similarly, for metabolic, endocrine, hematological and functional conditions, confirmatory testing, treatment supply chains, specialist referral and long-term monitoring must be considered before population-wide screening is introduced.

Finally, the framework underscores that technical readiness alone is insufficient. Sustainable financing, government stewardship, community engagement and family support are central to equitable implementation. Out-of-pocket payment models risk excluding the children most likely to benefit, while stigma and poor communication may reduce uptake and follow-up. The inclusion of affected persons and families in the consultation process reinforced the need for programmes that are not only clinically effective, but also acceptable, accessible and responsive to lived experience. As countries consider how to operationalize the WHA resolution, this framework can support a phased, context-specific approach that links NBS to the broader goals of universal health coverage, disability inclusion and child survival.

## 5. Conclusions

NBS and care for infants with birth defects can no longer remain peripheral to child health agendas in LMICs. The WHO framework provides a practical route for immediate action: start with one or a few feasible, high-impact conditions; link every positive screen to diagnosis, treatment, rehabilitation and follow-up; and build systems for progressive expansion. Ministries of health, partners and funders are encouraged to move rapidly from commitment to implementation by embedding birth defects services within universal health coverage, financing, referral and treatment pathways and ensuring that every newborn has access to screening, diagnosis, and timely, lifesaving and disability-preventing care.

## Figures and Tables

**Figure 1:**
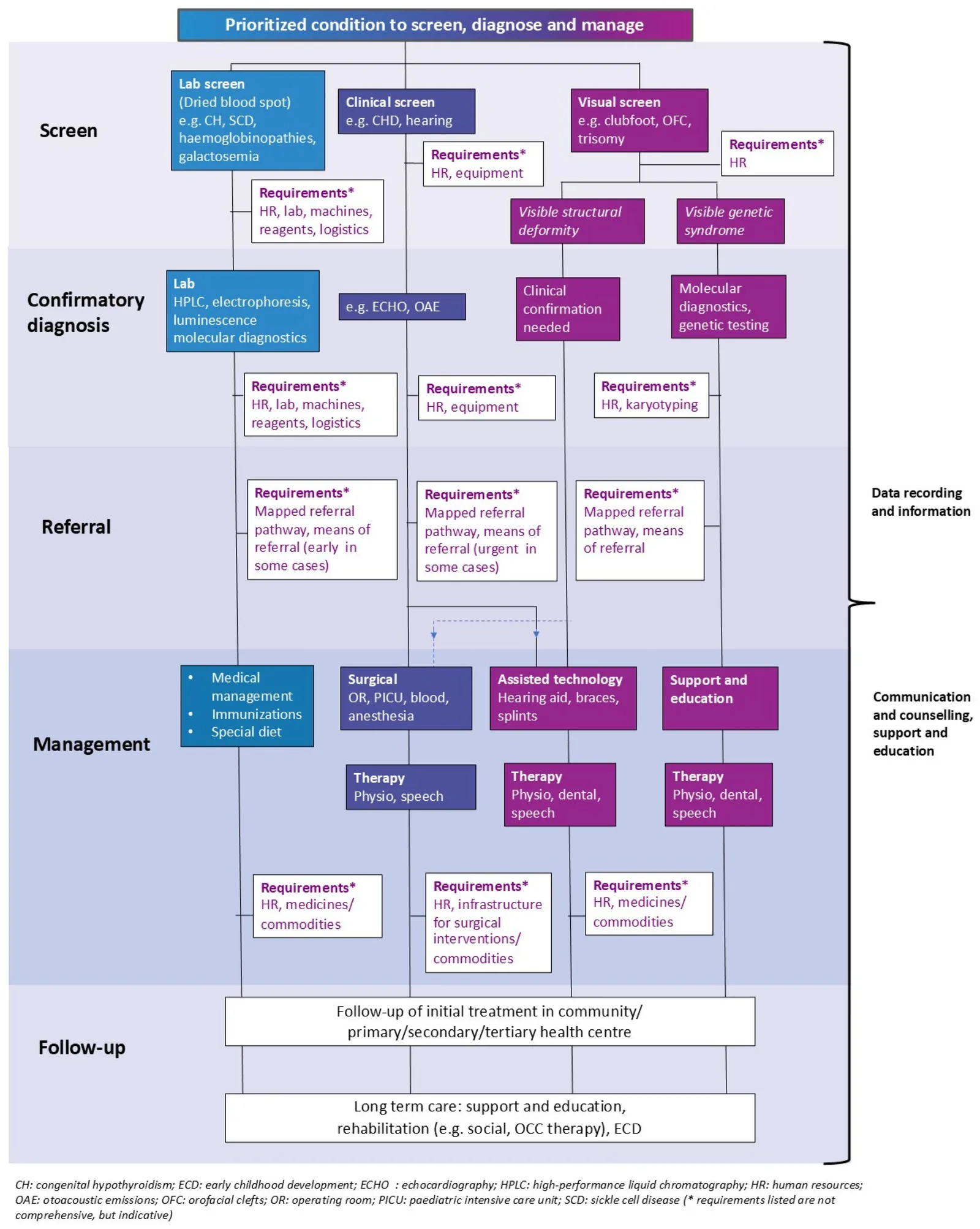
An operational framework to assess health system preparedness for newborn screening, diagnosis and management of a selected birth defect.

## Data Availability

No new data were created or analyzed in this study. Data sharing is not applicable to this article.

## References

[1] Center for Disease Control Prevention (2017). Morbidity and Mortality Weekly Report.

[2] Duan J., Ding R., Yu Y., Li M., Ruan Y., Hu Y., He Y., Sun Z. (2025). Global and regional burden of congenital birth defects, 1990–2021: Persistent healthcare disparities and emerging challenges from non-fatal health burden. BMJ Public Health.

[3] Christianson A., Howson C.P., Modell B. (2005). March of Dimes: Global Report on Birth Defects, the Hidden Toll of Dying and Disabled Children.

[4] Perin J., Mai C.T., De Costa A., Strong K., Diaz T., Blencowe H., Berry R.J., Williams J.L., Liu L. (2023). Systematic estimates of the global, regional and national under-5 mortality burden attributable to birth defects in 2000–2019: A summary of findings from the 2020 WHO estimates. BMJ Open.

[5] United Nations Inter-Agency Group for Child Mortality Estimation (2025). Levels and Trends in Child Mortality: Report 2025. https://data.unicef.org/resources/levels-and-trends-in-child-mortality-2025/.

[6] World Health Assembly (2024). Seventy-Seventh World Health Assembly: Geneva, 27 May–1 June 2024: Resolutions and Decisions, Annexes. https://iris.who.int/handle/10665/381061.

[7] World Health Organization (2026). Strengthening Capacity for Newborn Screening, Diagnosis and Management of Birth Defects: WHO Technical Consultation on Planning and Implementing Services Integrated into National Health Systems in Low- and Middle-Income Countries.

[8] González Reyes E.C., Castells E.M., Frómeta A., Arteaga A.L., Del Rio L., Tejeda Y., Perez L.T., Segura M.T., Almenares P., Perea Y. (2016). SUMA technology and newborn screening tests for inherited metabolic diseases in Cuba: An overview of the first 30 years. J. Inborn Errors Metabol. Screen..

[9] Bidondo M.P., Groisman B., Barbero P., Liascovich R. (2015). Public health approach to birth defects: The Argentine experience. J. Community Genet..

[10] Padilla C.D., Therrell B.L., Alcausin M.M.L.B., Chiong M.A.D., Abacan M.A.R., Reyes M.E.L., Jomento C.M., Dizon-Escoreal M.T.T., Canlas M.A.E., Abadingo M.E. (2022). Successful implementation of expanded newborn screening in the Philippines using tandem mass spectrometry. Int. J. Neonatal Screen..

[11] Chaube P., Singh A.K., Choudhury M.C. (2023). Expansion of India’s national child healthcare programme, Rashtriya Bal Swasthya Karyakram (RBSK), for rare disease management: A health policy perspective. Orphanet J. Rare Dis..

[12] Hernandez A.G., Kiyaga C., Howard T.A., Ssewanyana I., Ndeezi G., Aceng J.R., Ware R.E. (2021). Operational analysis of the national sickle cell screening programme in the Republic of Uganda. Afr. J. Lab. Med..

[13] Smythe T., Owen R.M., Aspden A., Everhart J., Abera E., Amaraegbulam P., Flores R., Valdez L., Lavy C. (2025). Global clubfoot treatment in 2023: An overview of advances and outcomes. BMJ Glob. Health.

[14] Nair S.M., Zheleva B., Dobrzycka A., Hesslein P., Sadanandan R., Kumar R.K. (2021). A population health approach to address the burden of congenital heart disease in Kerala, India. Glob. Heart.

[15] Karma Law District Early Intervention Centres: Nurturing Child Development in India. https://karma.law/insights/law-library/district-early-intervention-centres-nurturing-child-development-in-india/.

[16] Padilla C.D., Abadingo M.E., Munda K.V., Therrell B.L. (2023). Overcoming challenges in sustaining newborn screening in low-middle-income countries: The Philippine newborn screening system. Rare Dis. Orphan Drugs J..

[17] Republic of the Philippines (2004). Newborn Screening Act of 2004, Republic Act No. 9288. https://www.lawphil.net/statutes/repacts/ra2004/ra_9288_2004.html.

[18] Caneo L.F., Miana L.A., Garros D., Neirotti R. (2022). A new dawn for Brazilian pediatric cardiac surgery is on the way-issues around and outside the operating room. Braz. J. Cardiovasc. Surg..

[19] Global PaedSurg Research Collaboration (2021). Mortality from gastrointestinal congenital anomalies at 264 hospitals in 74 low-income, middle-income, and high-income countries: A multicentre, international, prospective cohort study. Lancet.

[20] Therrell B.L., Padilla C.D. (2014). Barriers to implementing sustainable national newborn screening in developing health systems. Int. J. Pediatr. Adolesc. Med..

[21] Folayan O.S., Orimadegun B.E., Ayede A.I., Inusa B.P., Kase M.K., Anetor J.I. (2026). Integrated Newborn Screening in Nigeria: The Way Forward, A Workshop Report. Int. J. Neonatal Screen..

